# Minimally Invasive Surgical Approach for Parotid Sialolith Removal

**DOI:** 10.7759/cureus.87393

**Published:** 2025-07-06

**Authors:** Subhasish Burman, Asish K Das, Diptangshu Mallick, Sandhya Tamilmani, Samrat Hembram

**Affiliations:** 1 Oral and Maxillofacial Surgery, Dr. R. Ahmed Dental College and Hospital, Kolkata, IND

**Keywords:** minimally invasive surgery, parotid duct calculus, parotid sialolithiasis, salivary gland stones, sialolithiasis treatment

## Abstract

Sialolithiasis is one of the most common diseases that affects the salivary glands and develops stones inside the salivary glands. We describe the case of a 36-year-old man who had been experiencing pain and swelling in the left cheek area for a month, along with discharge in the left posterior buccal vestibular region. A 3.5 mm calculus in the major parotid duct was discovered during the ultrasound scan. Under general anesthesia, the patient had a superficial parotidectomy, and the calculus was successfully removed. This case study illustrates the value of early detection and treatment of parotid sialolithiasis and shows how well superficial parotidectomy works to treat the problem.

## Introduction

Sialolithiasis (term sialo meaning salivary and lithiasis meaning formation of stone or calcareous concretions) is one of the most common diseases that affect the salivary glands and ultimately leads to dysfunction of the salivary gland [1,2]. Another term salivary duct lithiasis is characterized by obstruction of the excretory duct due to sialoliths [3,4]. Sialolith mostly occurs in the submandibular gland (80% to 92%) due to its position and secretion against gravity. In the parotid gland, the rate of occurrence of sialolith is 6% to 20%, and sublingual glands and other minor glands comprise 1% to 2% [5,6], presenting with symptoms of pain and swelling, called the meal-time syndrome [4,7].

In 1896, Küttner did a clinical study and proposed that chronic sialadenitis is the primary condition that leads to the formation of sialolith [8]. Later on, this proposal was not accepted [9].

Sialadenitis is the inflammation of the salivary gland. It may be due to bacterial infection, autoimmune diseases, IgG4-related diseases, or granulomatous diseases. The parotid gland is prone to bacterial sialadenitis because it has fewer lysozymes, lower concentrations of secretory immunoglobulin A (IgA), and sialic acids, which inhibit bacterial adherence by causing agglutination of bacteria and glycoproteins [10].

Several hypotheses have been put forward to explain the etiology of the calculi. The combination of mechanical, inflammatory, chemical, neurogenic, infectious, strange bodies, etc., initiates the precipitation of the amorphous tricalcic phosphate and transforms into hydroxyapatite, along with traces of magnesium, carbonate, and ammonia [1,3]. Another theorem states that, due to long-term stagnation of saliva, gel-like material will form because mucoid changes occur in saliva content, which helps in the aggregation of organic and inorganic substances [2,10]. The exact mechanism is still mystifying.

The parotid is a serous gland. Histologically, the parotid rarely has mucous acini and mucous goblet cells that contain acidic mucin, which are found in the lining of collecting ducts [11].

In the parotid glands, calculi predominantly occur unilaterally, are smaller, are single or multiple, and are located within the ductal system and rarely in the intraparenchymal of the gland [2,3], with predominance in the fourth decade and male predilection [1,2].

There are many differential diagnoses related to salivary gland pathology. This can be done by taking a proper case history, doing clinical examination and investigation like plain radiograph, ultrasonography, sialography, MR sialography, CT scan, FNAC, and diagnostic sialo-endoscopy, according to the need [2,3,10]. Here, we are going to discuss a case report in which a long-standing parotid sialolith leads to chronic sialadenitis.

## Case presentation

**Figure 1:**
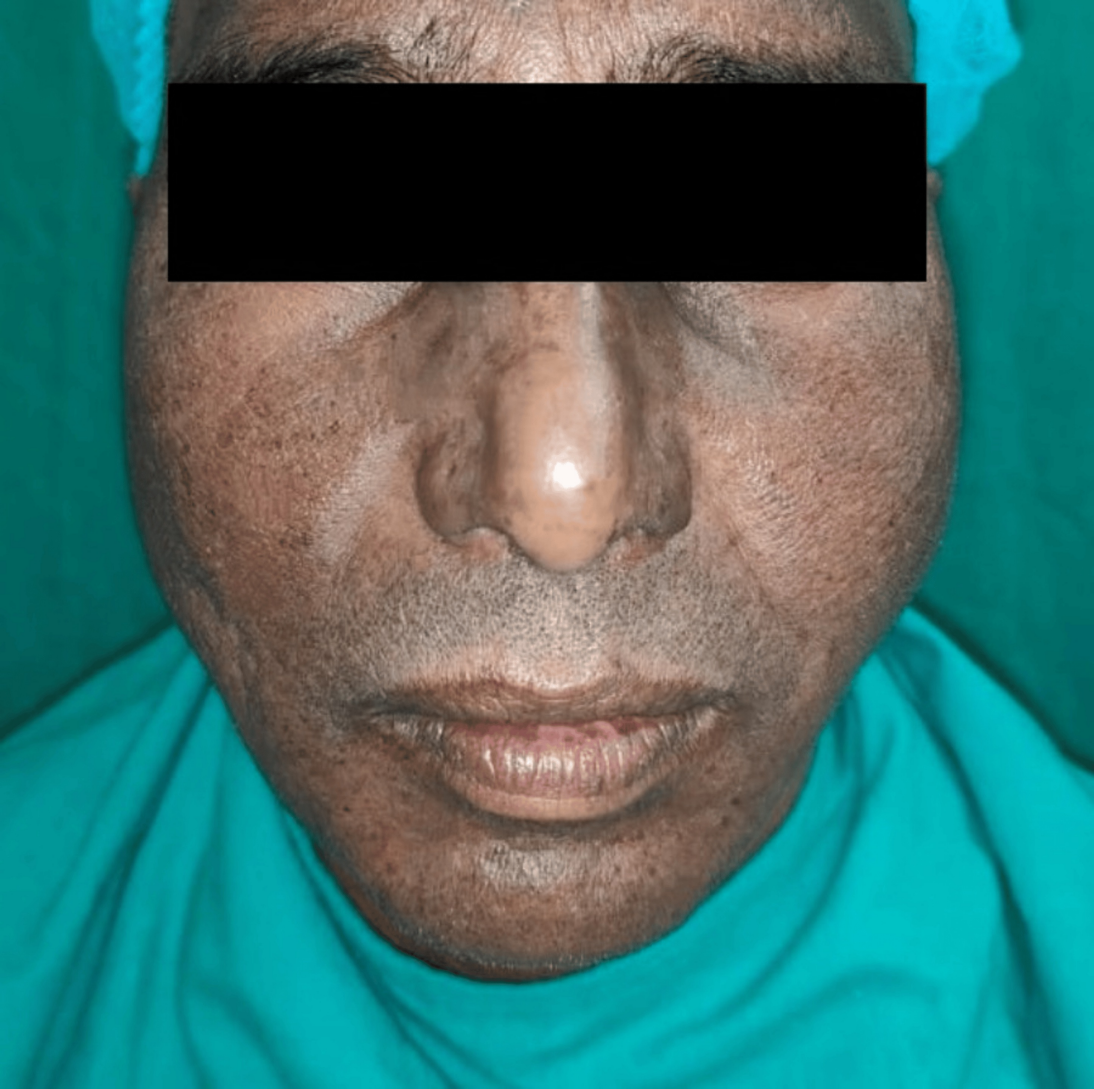
Preoperative extraoral frontal view Image showing asymmetry of the face. Swelling on the left side of the face

**Figure 2:**
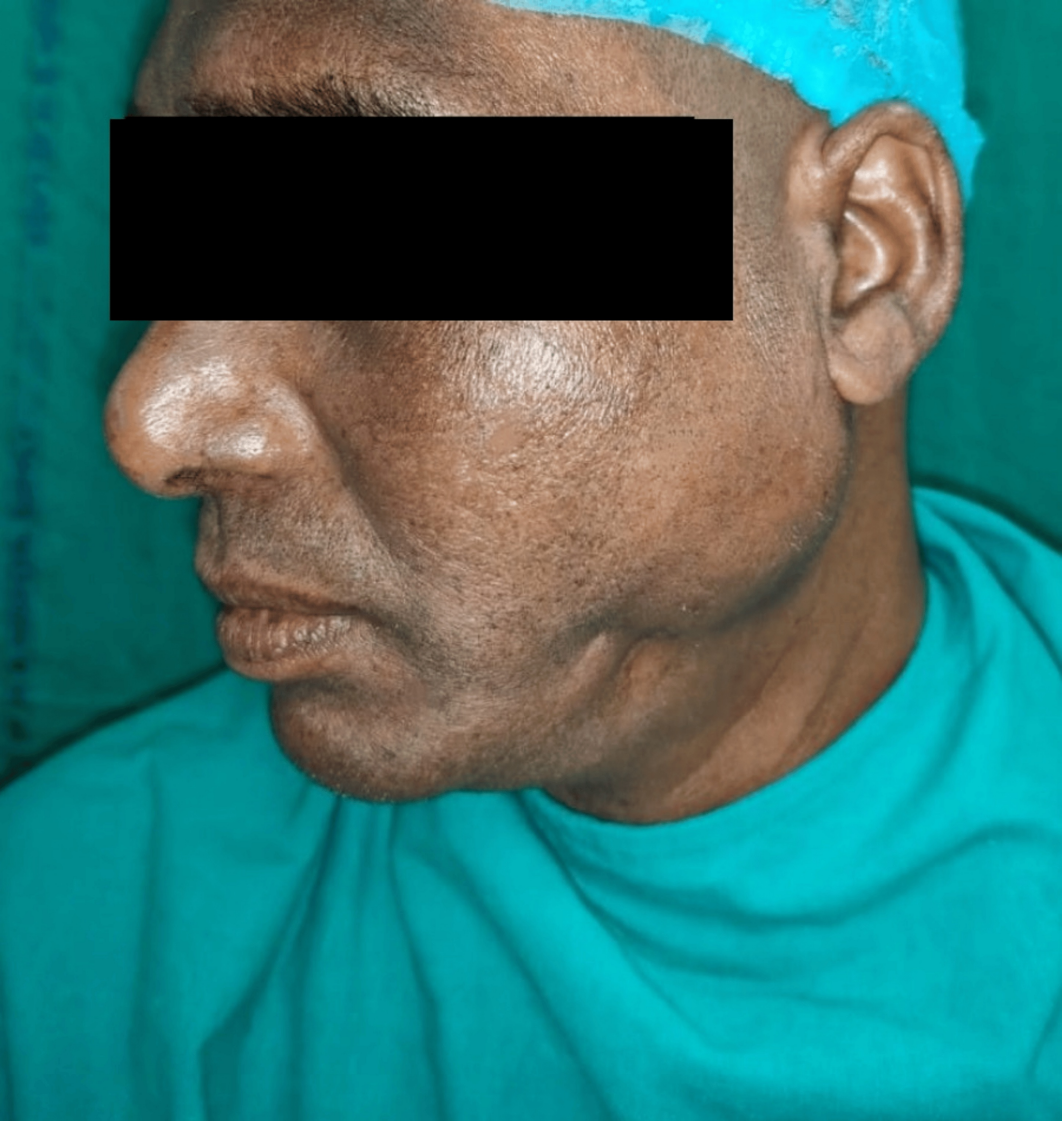
Preoperative extraoral photograph showing swelling on the left side of the face Swelling extended anteroposteriorly from the zygomatic buttress to the mastoid region and superoinferiorly from the tragus to the inferior border of the mandible

A 36-year-old male patient attended the Department of Oral and Maxillofacial Surgery of Dr. R. Ahmed Dental College and Hospital, with a swelling over the left cheek region, in front of the ear, for the last month. The swelling was associated with pain which was dull, throbbing, and radiating to the ear, and used to increase during meal time. There was also a history of discharge intraorally on the left posterior region. Past medical and dental history were insignificant. On extraoral examination, the swelling extended anteroposteriorly from the zygomatic buttress to the mastoid region and superoinferiorly from the tragus to the inferior border of the mandible and was about 4 × 3 cm in dimension (Figures 1, 2).

**Figure 3:**
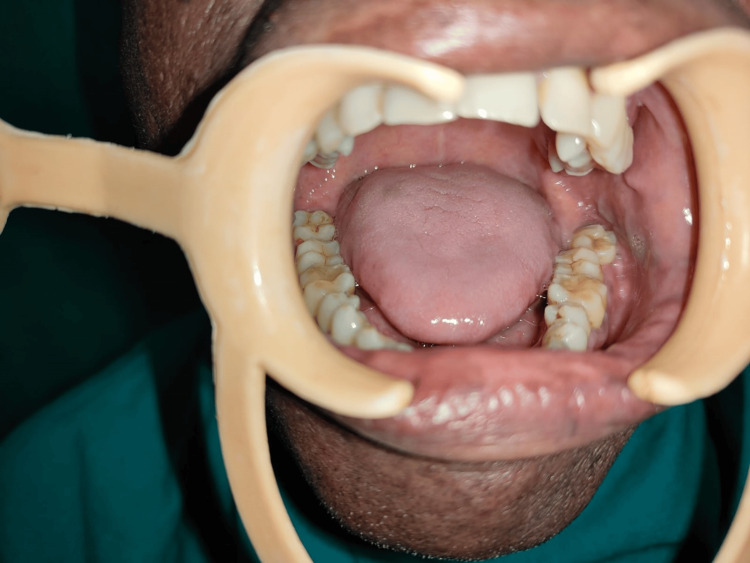
Intraoral photograph showing no anomaly The intraoral picture showing nothing significant. Normal anatomy of the mouth

On palpation, the swelling was nontender and firm in consistency. No lymph nodes were palpable. There was no evidence of any involvement of facial nerve or muscle weakness. Intraoral findings were clinically insignificant (Figure 3).

**Figure 4:**
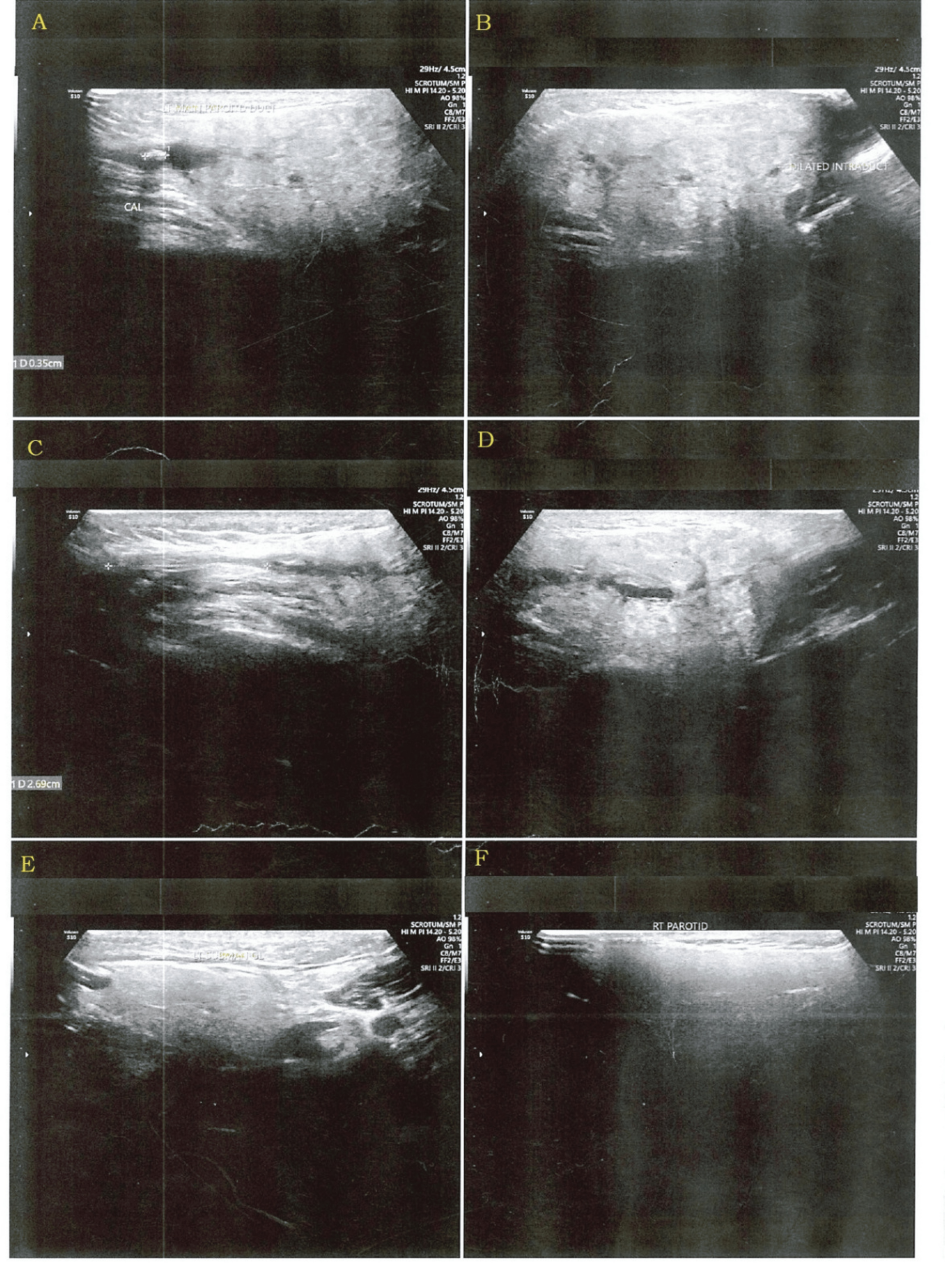
USG showing calculi USG: ultrasonography There are six boxes in the image. Each box is labeled as A, B, C, D, E, and F. A shows the calculi. The USG shows the main parotid duct calculi

On ultrasonography, the parotid duct showed a calculus of size of 3.5 mm, in the lumen which was about 2.7 mm away from the drainage site (Figure 4).

**Figure 5:**
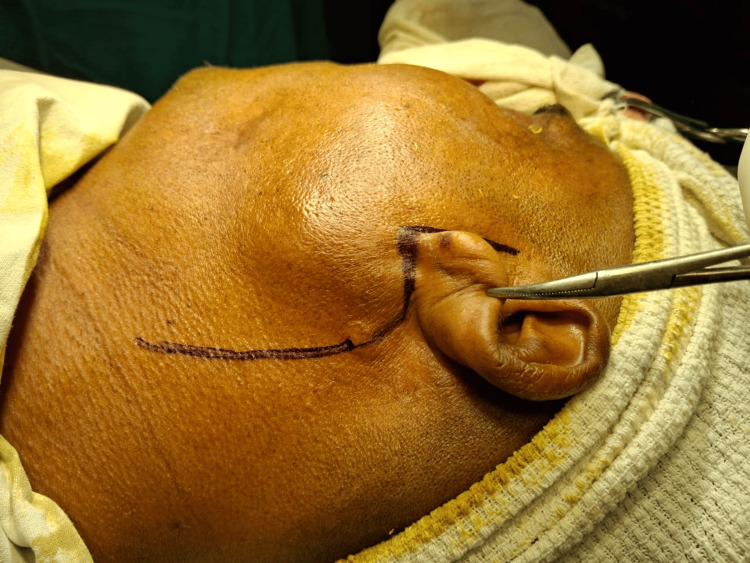
Incision marked and a modified Blair incision was made The modified Blair incision was made over the preauricular skin crease, beginning from the anterior and superior to the tragus and carried down to the left earlobe. It was extended posteriorly to the postauricular area and then again extended inferiorly to the naturally occurring horizontal skin crease on the neck

**Figure 6:**
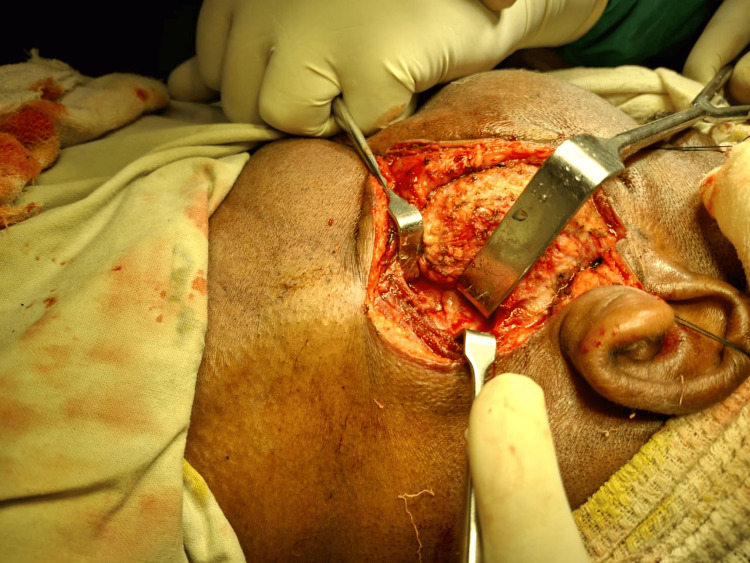
Exposure of the parotidomasseteric fascia

We arrived at a provisional diagnosis from the radiological features and clinical evaluation to be left-sided sialolithiasis with chronic sialadenitis. The treatment plan was superficial parotidectomy under general anesthesia. The patient was intubated uneventfully, scrubbed, and draped. The incision was marked and infiltrated with diluted adrenaline solution. A modified Blair incision was done, and a layer-by-layer dissection was done to expose the parotidomasseteric fascia (Figures 5, 6).

**Figure 7:**
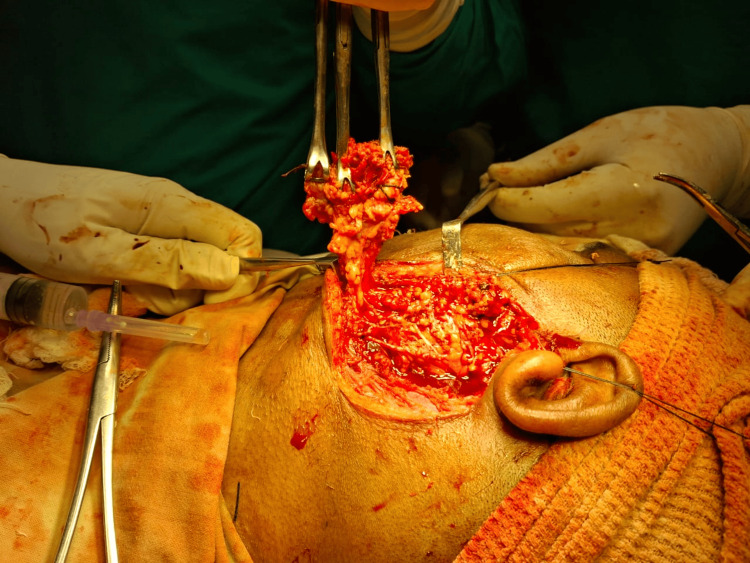
Superficial lobe of the parotid gland Three Babcock forceps have been used to hold the superficial lobe of the parotid

The modified Blair incision was made over the preauricular skin crease, beginning from the anterior and superior to the tragus, and carried down to the left earlobe. It was extended posteriorly to the postauricular area and then again extended inferiorly to a naturally occurring horizontal skin crease on the neck. After incising the skin and platysma, an anterior skin flap is raised in a plane between the superficial musculoaponeurotic system (SMAS) and the superficial capsule of the parotid. The superficial lobe of the parotid gland was identified (Figure 7).

**Figure 8:**
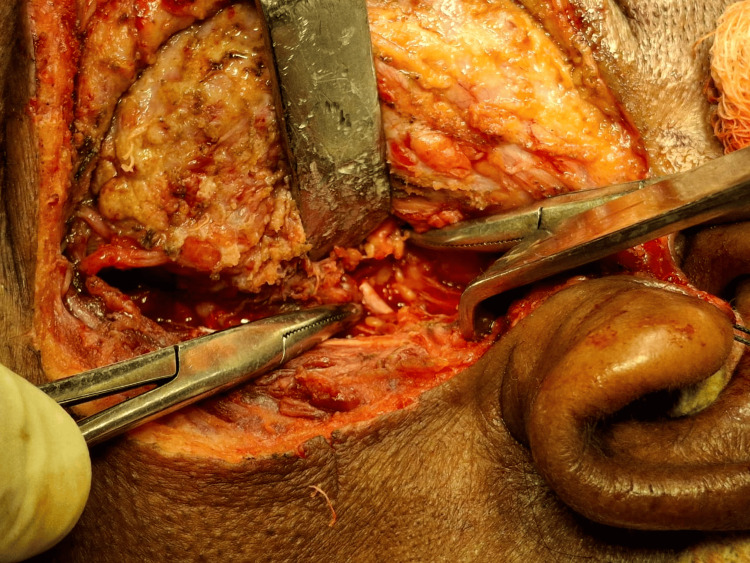
Main trunk of the facial nerve Artery forceps showing the main trunk ofthe  facial nerve

The facial nerve was identified and checked with a nerve stimulator (Figure 8).

**Figure 9:**
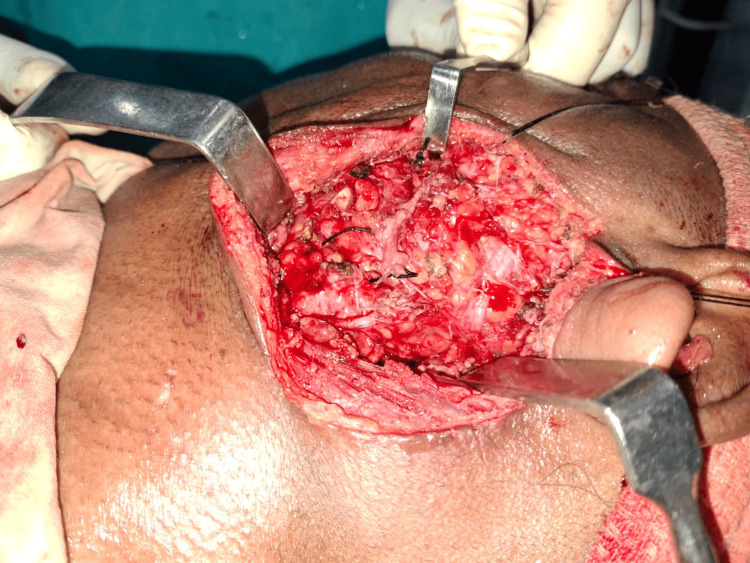
Removal of the superficial lobe The superficial parotid gland has been removed. Hemostasis has been achieved

The superficial lobe of the parotid gland was dissected and separated from the facial nerve (Figure 9).

**Figure 10:**
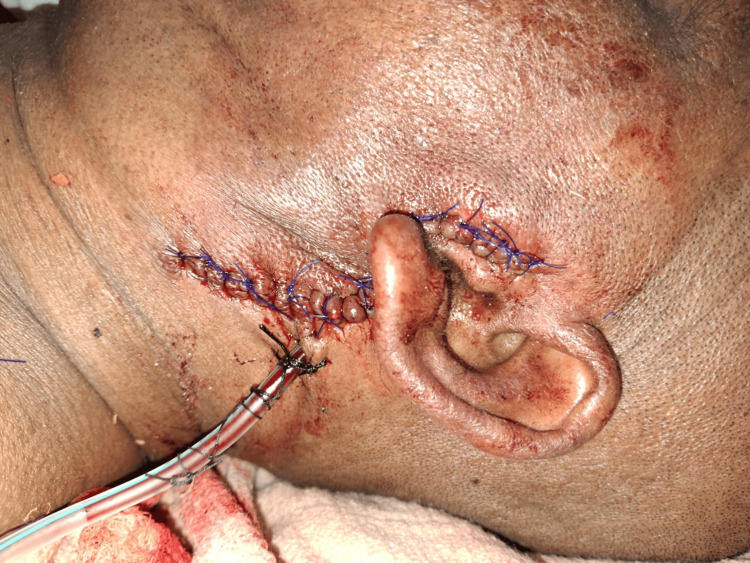
Suturing done and drain has been placed Extraoral suturing done with 3-0 Prolene. A 14 G drain has been placed

The parotid duct and the calculus were identified and transected. The remaining duct was ligated using 2-0 Vicryl. The parotidomasseteric fascia was sutured using 2-0 Vicryl. A 14 G drain was placed. A layer-by-layer closure was done with 2-0 Vicryl. Skin sutures were given using 3-0 Prolene (Figure 10).

**Figure 11:**
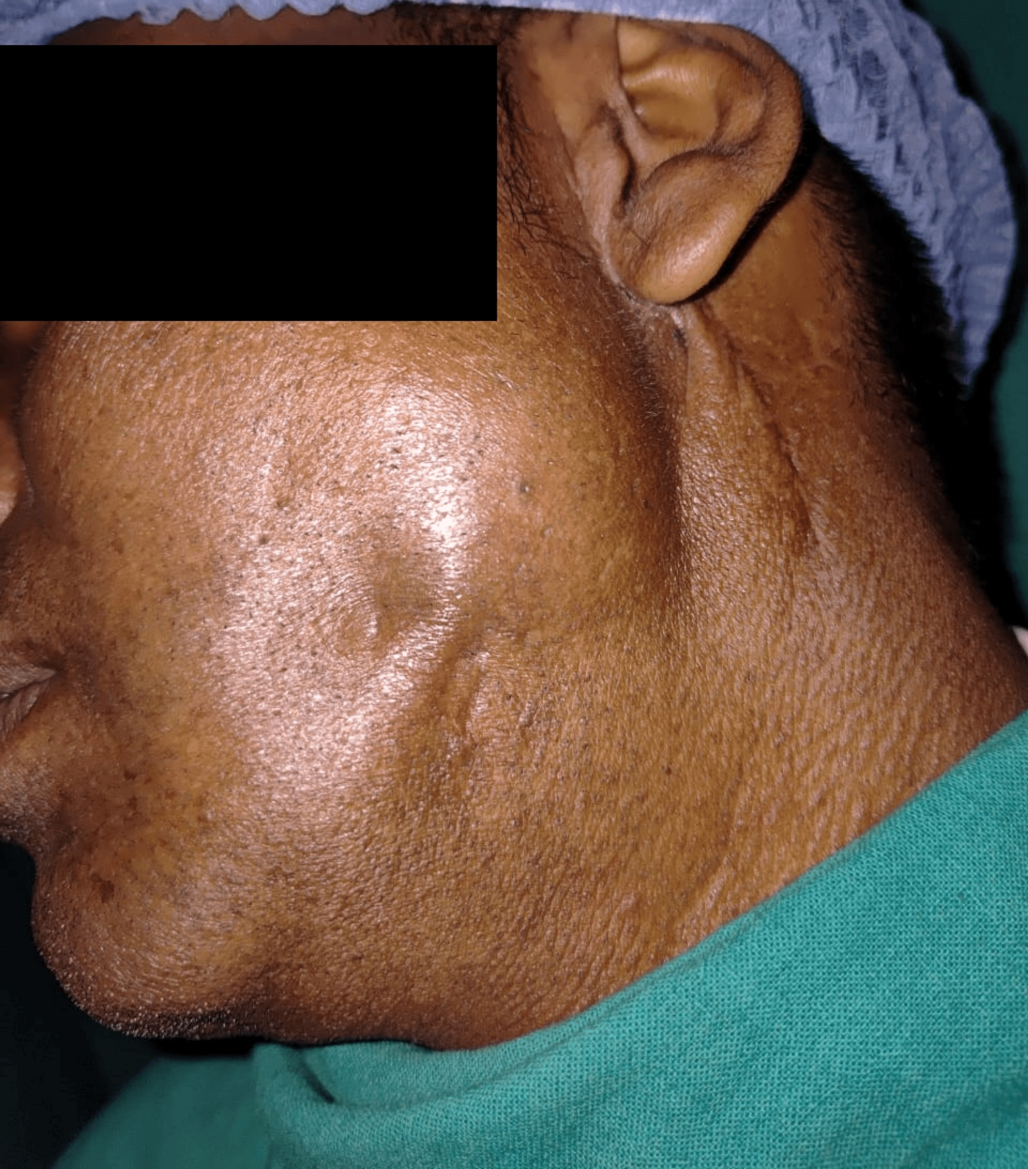
Postoperative picture after one-month follow-up Postoperative follow-up after one month showing that swelling had reduced in size, and wound healed uneventfully No discharging sinus tract is present

During postoperative follow-up, the patient was examined for facial nerve dysfunction, which was absent. Postoperative healing was uneventful (Figure 11).

**Figure 12:**
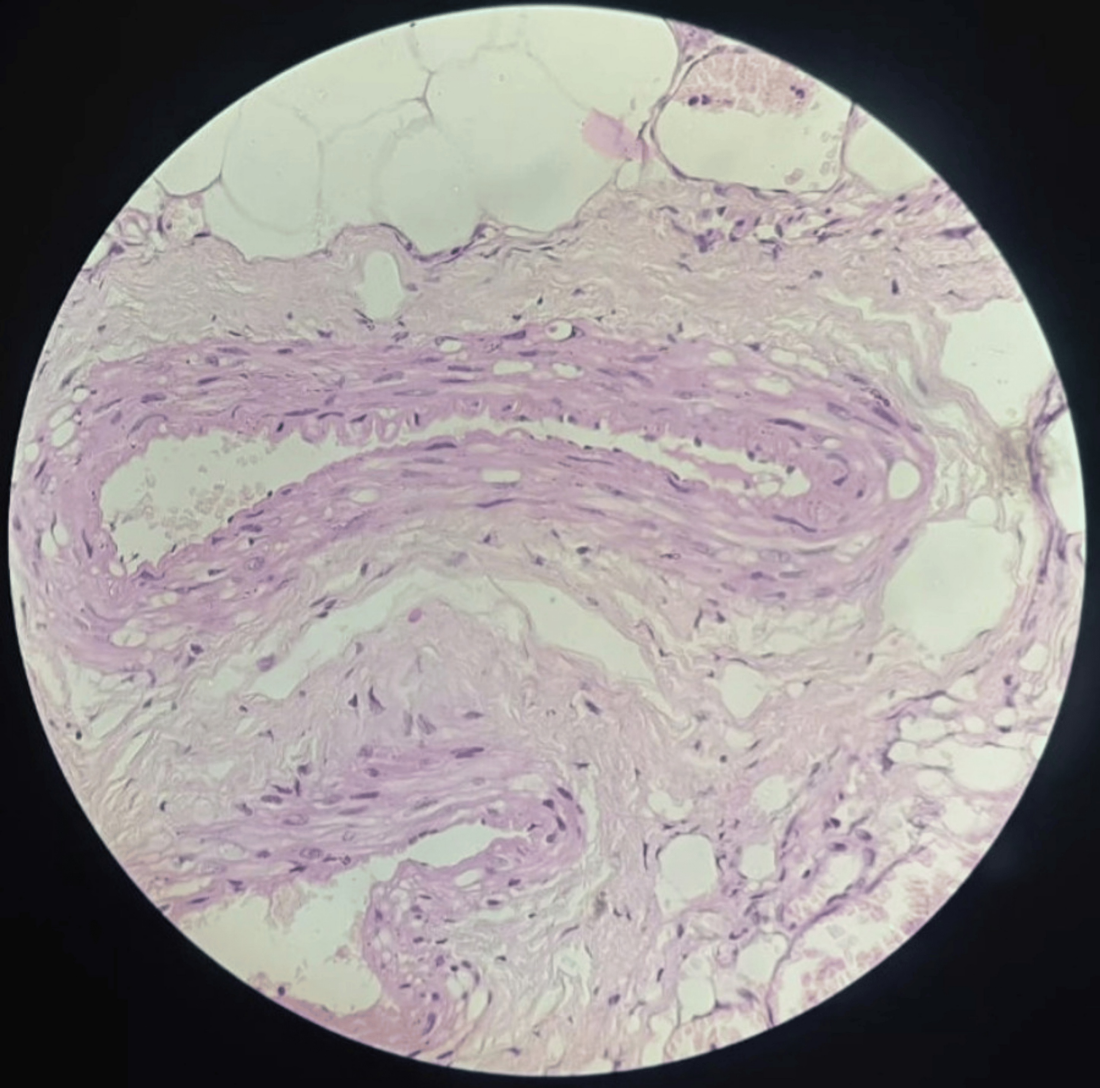
Histopathological evaluation There is degeneration of the acini with effacement of the lobular architecture and the presence of epimyoepithelial islands and adipose tissue along with diffuse inflammatory cell infiltrates

The histopathological analysis revealed a single bit of soft tissue which is composed of encapsulated salivary gland acini with fibrous septate between the lobes and lobules. There is degeneration of the acini with effacement of the lobular architecture and the presence of epimyoepithelial islands and adipose tissue along with diffuse inflammatory cell infiltrates (Figure 12).

## Discussion

The largest salivary gland is the parotid gland. The masseter muscle forms the gland's anterior border, followed by the external auditory canal on the back, the zygomatic arch on the top, and the anteromedial aspect of the sternocleidomastoid muscle on the bottom. The gland is essentially divided into superficial and deep lobes by the facial nerve. The facial nerve and the deep lobe extend medially into the parapharyngeal region, while the superficial lobe is lateral. Since there is no real embryologic or fascial plane between the pars superficialis and pars profunda, this division is not anatomical. The deep cervical fascia, which separates into a deep and superficial layer to enclose the gland, prolongs around the parotid gland. From the masseter and sternocleidomastoid to the zygomatic arch, the superficial fascia runs. The platysma to the superficial temporal fascia is the superior extension of the SMAS. The parotid fascia is superficial to the SMAS [12,13].

The parotid duct, also known as the Stenson duct, leaves the gland anteriorly and travels parallel to the zygomatic arch and about 1 cm inferior to it. The duct enters the oral cavity, facing the second maxillary tooth, after passing through the masseter and puncturing the buccinator muscle. In contrast to the submandibular gland, which secretes a more mixed serous/mucous saliva, the parotid gland produces more serous saliva. This explains why sialolithiasis is more common in the submandibular gland [14,15]. The most common condition affecting the major and minor salivary glands is sialoliths, which are calcium salt condensations. Both males and females are equally affected [16].

Numerous reasons, such as decreased crystalloid solubility, excessive alkalinity, elevated calcium content, physical damage to the salivary duct or gland, and oropharyngeal infection, can cause decreased saliva production, dehydration, and a shift in salivary pH. It has been demonstrated that systemic conditions including gout, smoking, and the use of diuretics all contribute to the development of calculi. In salivary stones, inorganic or mineral salts in the form of hydroxyapatite, whitlockite, and brushite have precipitated along with organic components such as bacteria or desquamated cells, glycoproteins, collagen, lipids, other proteins, and carbohydrates [17].

A thorough medical history and physical examination must be the first steps in the assessment of a patient with salivary gland enlargement. Carlson, in his article “Diagnosis and Management of Salivary Gland Infections,” mentioned a detailed step-by-step diagnostic algorithm for a parotid swelling. In this article, they have mentioned both bilateral and unilateral swelling and the role of clinical examination and radiographs, also [18].

The 36-year-old male patient in this case report had been experiencing pain and swelling in the left cheek area for a month, along with discharge in the left posterior buccal vestibular region. A 3.5 mm calculus in the major parotid duct was discovered during the ultrasound scan.

Iro et al. in their article “Current Concepts in Diagnosis and Treatment of Sialolithiasis” have mentioned the treatment algorithm. In their article, they mentioned the role of extracorporeal lithotripsy in removing salivary stones [19].

Under general anesthesia, the patient had a superficial parotidectomy, and the calculus was successfully removed. Imaging tests including ultrasound, CT, and MRI might be useful in verifying the diagnosis of parotid sialolithiasis, which can be difficult to diagnose. In this instance, the calculus was found and surgical therapy was guided by the results of the ultrasound test.

Sharma, in his article “Superficial Parotidectomy for Chronic Parotid Sialadenitis,” mentioned about 21 patients who underwent superficial parotidectomy for chronic parotid sialadenitis. Six patients (28.57%) developed temporary facial nerve palsy. Three (14.28%) patients developed Frey’s syndrome. Paresthesia of the ear lobe was found in all cases. In one case (4.76%), each of sialocoele and hypertrophic scar was found. There was a complete resolution of symptoms in all the cases [20].

Parotid sialolithiasis can be difficult to treat surgically, and the facial nerve and surrounding structures must be carefully taken into account. One popular method is superficial parotidectomy, which entails removing the parotid gland's superficial lobe. In this instance, the calculus was successfully removed during a superficial parotidectomy performed on the patient while under general anesthesia. The patient had a smooth postoperative recovery and was released from the hospital without any issues. This demonstrates the efficacy and safety of superficial parotidectomy in the treatment of parotid sialolithiasis.

## Conclusions

This case study shows how superficial parotidectomy is used to successfully treat a patient with parotid sialolithiasis. The surgery recovery was uneventful, and the patient's symptoms were successfully alleviated. This case demonstrates the safety and efficacy of superficial parotidectomy in the management of parotid sialolithiasis, as well as the significance of early identification and treatment. The effective resolution of this case adds to the body of knowledge already available on the treatment of parotid sialolithiasis and offers clinicians handling related situations a useful educational opportunity.
